# Elevated Serum Levels of Mixed Lineage Kinase Domain-Like Protein Predict Survival of Patients during Intensive Care Unit Treatment

**DOI:** 10.1155/2018/1983421

**Published:** 2018-02-11

**Authors:** Mihael Vucur, Christoph Roderburg, Lukas Kaiser, Anne Theres Schneider, Sanchari Roy, Sven Heiko Loosen, Mark Luedde, Christian Trautwein, Alexander Koch, Frank Tacke, Tom Luedde

**Affiliations:** ^1^Department of Medicine III, University Hospital Aachen, RWTH Aachen University, Pauwelsstrasse 30, 52074 Aachen, Germany; ^2^Division of GI and Hepatobiliary Oncology, University Hospital Aachen, RWTH Aachen University, Pauwelsstrasse 30, 52074 Aachen, Germany; ^3^Department of Internal Medicine III, University of Kiel, Rosalind-Franklin-Str. 12, 24105 Kiel, Germany

## Abstract

Mixed lineage kinase domain-like (MLKL), a crucial regulator of necroptotic cell death, was shown to play a role in inflammatory diseases. However, its role as a biomarker in critical illness and sepsis is currently unknown. We analyzed serum levels of MLKL in 136 critically ill patients at admission to the intensive care unit (ICU) and after three days of ICU treatment. Results were compared with 36 healthy controls and correlated with clinical and laboratory patients' data. MLKL serum levels of critically ill patients at admission to the ICU were similar compared to healthy controls. At ICU admission, MLKL serum concentrations were independent of disease severity, presence of sepsis, and etiology of critical illness. In contrast, median serum levels of MLKL after three days of ICU treatment were significantly lower compared to those at admission to the ICU. While serum levels of MLKL at admission were not predictive for short-term survival during ICU treatment, elevated MLKL concentrations at day three were an independent negative predictor of patients' ICU survival. Thus, elevated MLKL levels after three days of ICU treatment were predictive for patients' mortality, indicating that sustained deregulated cell death is associated with an adverse prognosis in critical illness.

## 1. Introduction

In the last years, our understanding of how cell death processes are involved in the pathophysiology of inflammatory and infectious diseases has been drastically altered. Besides apoptosis, multiple forms of regulated necrosis have been associated with pathologies such as diabetes, nonalcoholic steatohepatitis (NASH), heart failure, neurodegenerative diseases, and cancer [1–6].

Necroptosis results from oligomerization of mixed lineage kinase domain-like protein (MLKL) [7], which is initiated by receptor-interacting serine/threonine-protein kinase 3- (RIPK3-) dependent phosphorylation [8, 9]. MLKL then forms a pore that leaks intracellular contents, such as cytokines, chemokines, and other intracellular proteins [10]. Thus, the consequences of necroptosis are not necessarily proinflammatory. During bacterial infections, necroptosis can either promote pathogen removal or contribute to host pathology [11]. Moreover, it was recently demonstrated that necroptosis plays a fundamental role to limit overwhelming systemic inflammation in the context of *Staphylococcus aureus* sepsis [12]. MLKL as the key driver of necroptotic cell death might therefore represent an important regulator of sepsis as the most severe consequence of bacterial infections. It was shown in models of skin infection or sepsis that *Mlkl^−/−^* mice had high bacterial loads, an inability to limit interleukin-1*β* (IL-1*β*) production, and excessive inflammation [12]. In contrast, other groups demonstrated that *Mlkl^−/−^* mice were protected from severe pneumonia, highlighting the need for further research in this field [11].

Intracellular MLKL is the driving force behind necrotic cell death in many diseases. However, very little data is available on the potential role of circulating MLKL as a biomarker. In this study, we analyzed concentrations of circulating MLKL in a large cohort of patients treated in an intensive care unit (ICU). Most importantly, we show that elevated MLKL levels are predictive of impaired patients' survival after three days of ICU treatment, suggesting that MLKL represents a potential biomarker in patients with critical illness.

## 2. Materials and Methods

### 2.1. Study Design and Patient Characteristics

In the present study, we included 136 critically ill patients that were consecutively admitted to our medical ICU. Of those, 76 were male and 60 female. Patients' characteristics are presented in Table 1. The study protocol was approved by the local ethics committee (ethics committee of the University Hospital Aachen, RWTH Aachen University, Aachen, Germany) and conducted in accordance with the ethical standards laid down in the Declaration of Helsinki. Written informed consent was obtained from the patient, his or her spouse, or the appointed legal guardian. Patients' information and samples were acquired prospectively. Follow-up was performed as recently described [13]. Presence of sepsis disease was defined according to the criteria defined in the third consensus definitions of sepsis [14]. All other patients were categorized as nonsepsis patients [13, 15].

### 2.2. Measurements of MLKL Serum Levels by ELISA

Blood was collected at the time point of admission to the ICU and after 3 days of treatment. Sample handling and analysis of routine laboratory and experimental parameters were described previously [15–17]. MLKL serum levels were measured using a commercially available enzyme immunoassay according to manufacturers' instructions (MBS9300811, MyBioSource Inc.).

### 2.3. Statistical Analysis

Statistics applied in this analysis have been described in detail [15–18]. In summary, data are given as median and range. The Mann–Whitney *U* test or the Kruskal-Wallis ANOVA was used. Box plot graphics displays a statistical summary of the median, quartiles, range, and extreme values. Correlation analyses were performed by using Spearman correlation tests. The prognostic value of the variables was tested by univariate and multivariate analysis in the Cox regression model. Kaplan-Meier curves were plotted to display the impact on survival. All statistical analyses were performed with SPSS (SPSS, Chicago, IL, USA) [19, 20].

## 3. Results

### 3.1. MLKL Serum Concentrations in Critically Ill and Septic Patients at Admission to the ICU

**Figure 1:**
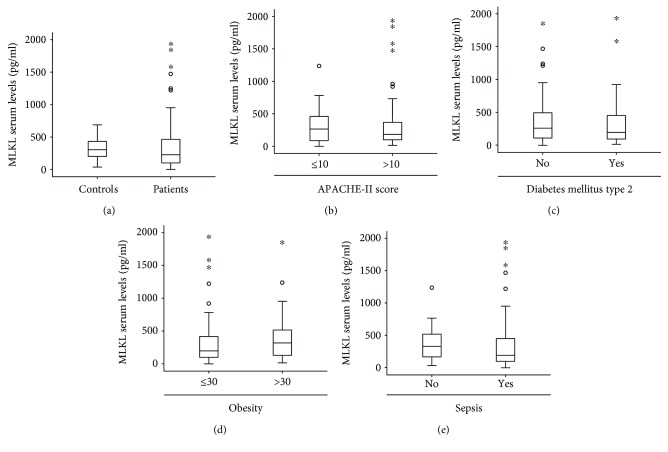
Serum levels of MLKL in critically ill patients at admission to the intensive care unit. (a) Serum concentrations of MLKL were analyzed by ELISA in critically ill patients at admission to the ICU and healthy blood donors as controls. (b) MLKL levels were analyzed with respect to the disease severity according to the APACHE-II score. (c) MLKL levels were analyzed with respect to the presence of type 2 diabetes. (d) MLKL levels were analyzed with respect to the presence of obesity (BMI > 30 kg/m^2^). (e) MLKL serum levels were analyzed in patients with sepsis and patients that did not fulfill the sepsis criteria. Asterisks and open circles indicate outlier values.

In order to prove the traceability of MLKL in human serum, we analyzed serum levels of MLKL by using ELISA in the serum of healthy blood donors and patients with different inflammatory or malignant diseases. Of note, we were able to reliably detect MLKL in the serum in all patients collectively (Supplementary Figure 1). To further explore a potential role of serum MLKL concentrations as a biomarker in critically ill and septic patients, we measured MLKL serum levels in a large and well-characterized cohort of 136 critically ill patients at admission to the ICU (patients' characteristics are given in Table 1) and 36 healthy blood donors. As seen in Figure 1(a), no significant differences in MLKL levels were detected between critically ill patients and the respective controls (Figure 1(a)). Moreover, MLKL concentrations were not related to disease severity, as assessed by correlation with the APACHE-II score (Figure 1(b)), or the presence of concomitant metabolic diseases, which had been previously linked to elevated MLKL levels in non-ICU populations [21] (Figures 1(c) and 1(d)).

We next analyzed the potential influence of sepsis on MLKL concentrations. Notably, no differences in MLKL levels between patients with sepsis (*n* = 96) and patients that did not fulfill sepsis criteria became apparent (*n* = 40; Figure 1(e)). We also tested if serum MLKL concentrations could be specifically deregulated in certain disease etiologies in critically ill patients. Our cohort of patients consisted of 55 patients with pulmonary sepsis, 12 with abdominal sepsis, 6 with urogenital sepsis, and 23 with a different/unknown focus of sepsis disease. Moreover, 40 patients suffered from nonsepsis etiologies of critical illness (15 cardiopulmonary diseases, 9 decompensated liver cirrhosis, 4 acute pancreatitis, and 12 had another etiology). When comparing serum MLKL concentrations among these different groups, no differences became apparent (data not shown). Altogether, these data suggest that circulating MLKL serum levels did not generally discriminate between critically ill and septic patients versus healthy controls.

### 3.2. MLKL Serum Concentrations in Critically Ill Patients at Admission to the ICU Are Not Associated with Patients' Survival

**Figure 2:**
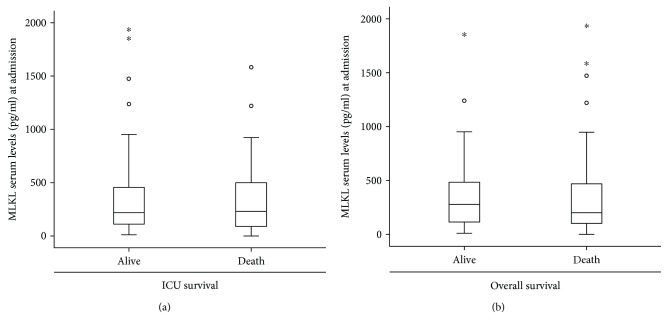
MLKL serum concentrations in critically ill at admission to the ICU are not associated with patients' survival. (a) Serum MLKL concentrations were measured in patients that died during ICU treatment and survivors. (b) MLKL serum concentrations were measured in patients that survived in the long-term follow-up and patients that did not survive. Asterisks and open circles indicate outlier values.

In order to explore MLKL as a prognostic biomarker, we assessed the potential association between circulating MLKL and patients' survival during ICU treatment (Figure 2(a)) or in their long-term follow-up (Figure 2(b)). We therefore subdivided our cohort of critically ill patients into survivors and patients that died during the respective observation periods. In line with the previous results, no differences in MLKL concentrations became apparent. Moreover, in a Cox regression analysis, MLKL was not an independent predictor for the patients' survival (data not shown), highlighting that MLKL serum levels at admission to ICU do not indicate the prognosis in critically ill patients.

### 3.3. MLKL Serum Concentrations after Three Days of ICU Treatment Predict Survival in Critically Ill Patients

**Figure 3:**
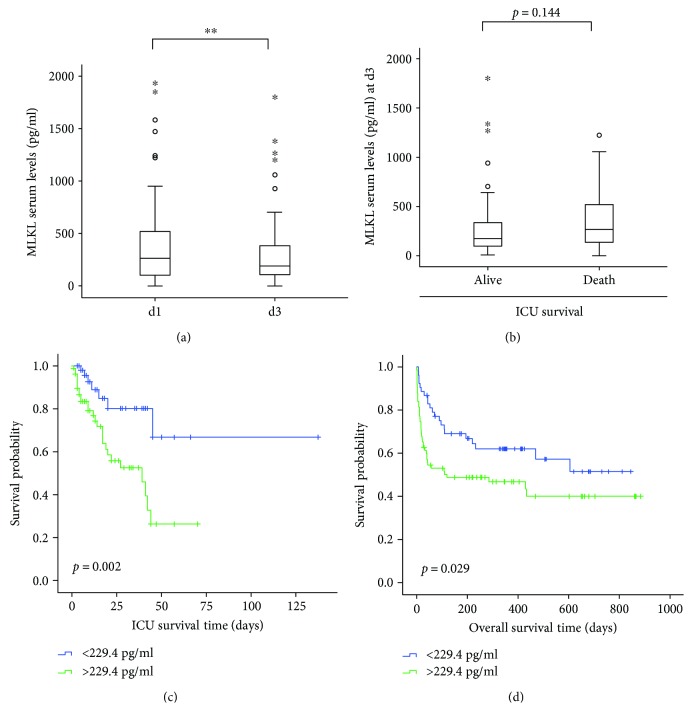
MLKL serum concentrations after three days of ICU treatment predict survival in critically ill patients. (a) Serum MLKL concentrations were measured in critically ill patients after three days of intensive care unit (ICU) treatment and compared to those measured at admission to the ICU, ^∗∗^ *p* < 0.01. Asterisks and open circles indicate outlier values. (b) Serum MLKL concentrations in critically ill patients were measured after three days of ICU treatment and analyzed with respect to patients' survival. Asterisks and open circles indicate outlier values. (c, d) Kaplan-Meier survival curves of ICU patients are displayed, showing that patients with high MLKL levels on day three (>229.4 pg/ml) had a significantly impaired survival at the ICU (c) or overall (d) as compared to patients with low MLKL serum concentrations (<229.4 pg/ml). The respective *p* values are given in the figure.

We and others recently demonstrated that longitudinal changes of serum markers during ICU treatment might be superior in detecting patients' prognosis compared to single biomarker concentrations measured at admission to the ICU [13]. We therefore compared serum concentrations of MLKL at admission to the ICU and those after three days of ICU treatment (d3; *n* = 93). Interestingly, serum levels of MLKL at d3 were significantly lower than those at admission to the ICU (Figure 3(a)). Moreover, in contrast to the results from the analysis at the time point of admission to the ICU, patients that survived ICU treatment demonstrated a clear trend towards lower MLKL serum concentrations compared to patients that succumbed to death (Figure 3(b)). Based on these results, we performed a Kaplan-Meier curve analysis and Cox regression analysis to examine the impact of MLKL levels on patients' survival in our cohort of critically ill patients. By using the Youden index [22], we first determined the optimal threshold for MLKL levels for prediction of ICU survival. This analysis revealed that MLKL serum concentrations of 229.4 pg/ml displayed the best sensitivity and specificity to predict patients' prognosis during ICU treatment. Using this cutoff, we performed Kaplan-Meier survival analysis, demonstrating that patients with MLKL concentrations > 229.4 pg/ml had a significantly impaired survival, while, in turn, patients with lower concentrations demonstrated a significantly more favorable prognosis (Figure 3(c)). Of note, the ICU mortality, within the “MLKL low” patients was 12.9% compared to 30.8% within the “MLKL high” patients (*p* = 0.004; Pearson chi-square test) based on MLKL serum measurement at day 3.

Within our group of critically ill patients, a total of 48.1% died, of which 25.7% died on the intensive care unit. By using Kaplan-Meier curve analysis, we tested whether MLKL serum levels (d3) at the cutoff described above are suitable to predict long-term survival in critically ill patients. Of note, this analysis revealed that, similar to ICU survival, patients with MLKL concentrations >229.4 pg/ml had a significantly impaired long-term survival (Figure 3(d)), strongly suggesting that persistently elevated levels of MLKL during ICU treatment indicate an unfavorable patients' prognosis. Notably, basal patients' characteristics (sex, presence of sepsis disease, etiology of sepsis disease, severity of sepsis disease, presence of liver cirrhosis, or diabetes mellitus type 2) were similar between both patient groups (Supplementary Table 1).

### 3.4. MLKL Serum Concentrations Correlate with Markers of Organ Failure in Critically Ill Patients

In order to identify factors regulating MLKL serum levels in patients with critical illness, we next applied correlation analyses between MLKL serum concentrations and a broad set of laboratory markers used in clinical routine on the ICU as well as experimental parameters. Interestingly, there were manifold correlations of serum MLKL concentrations at admission (d1) and at day 3 (d3) with surrogate markers of organ failure such as serum lactate concentrations (MLKL d1: *r* = 0.197, *p* = 0.028 and MLKL d3: *r* = 0.088, *p* = 0.427; Table 2), serum AST levels (MLKL d1: *r* = 0.279, *p* = 0.002 and MLKL d3: *r* = 0.225, *p* = 0.039; Table 2), and creatinine levels as a surrogate for an impaired kidney function (MLKL d1: *r* = 0.229, *p* = 0.008 and MLKL d3: *r* = 0.129, *p* = 0.220; Table 2). Notably, we also detected a close association between MLKL concentrations and suPAR (MLKL d1: *r* = 0.179, *p* = 0.042; Table 2), another experimental marker that has been demonstrated to indicate an impaired patient prognosis [23]. Finally, serum MLKL levels correlated with serum TNF concentrations (MLKL d1: *r* = 0.309, *p* = 0.044; Table 2), indicating biological plausibility of MLKL concentrations in patients with systemic inflammation.

## 4. Discussion

Here, we demonstrate that MLKL concentrations measured after three days of ICU treatment in critically ill patients predict prognosis during intensive care unit treatment. These data not only suggest a previously unrecognized function of MLKL as a biomarker in critical illness and sepsis but also highlight the clinical relevance of MLKL in the pathophysiology of inflammatory and infectious diseases.

MLKL has recently been identified as the key driver of necroptotic cell death. Necroptosis is a physiological cell suicide mechanism initiated by receptor RIPK3-dependent phosphorylation of MLKL, which results in disruption of the plasma membrane. Necroptotic cell lysis, and the resultant release of intracellular contents, is thought to cause inflammation in necroptotic disease models [21]. Several studies have investigated the role of necroptosis in sepsis and systemic inflammatory response syndrome (SIRS). Depending on the applied mouse and injury model, different results were observed. As such, studies on *Ripk3*-deficient mice showed protective effects in models of TNF-induced SIRS and sepsis [24, 25]. In contrast, investigations on *Mlkl*-deficient mice in the context of skin infection or gram-positive sepsis revealed that *Mlkl^−/−^* mice had higher bacterial loads, an inability to limit interleukin-1*β* (IL-1*β*) production, and excessive inflammation [12]. Again, other groups demonstrated that *Mlkl^−/−^* mice were protected from severe pneumonia, highlighting the need for further research to clarify this controversy [11].

In our study, serum levels of MLKL correlated with those of other proinflammatory cytokines such as TNF and suPAR, highlighting that elevated levels of MLKL might reflect the activation of immunological processes during sepsis disease (Table 2). Moreover, serum levels of MLKL correlated with established clinical markers of organ dysfunction and organ failure such as elevated AST/ALT levels and elevated serum bilirubin concentrations (Table 2). Sepsis-associated organ failure represents the consequence of circulatory failure and subsequent vasoconstriction leading to reduced blood flow and ischemic cell death. Recently, it was demonstrated that necroptosis is a key mediator of enterocytes loss in intestinal ischemia/reperfusion injury [2]. In line, we found a strong correlation of serum MLKL concentrations and serum lactate concentrations in our cohort of patients, supporting a link between MLKL and sepsis-associated organ failure (Table 2). Multiple organ failure represents the most important cause of mortality in critically ill and septic patients. Interestingly, serum levels of MLKL were lower in patients that displayed long time survival compared to patients that succumbed to death. The striking fact that patients with persistently elevated MLKL levels (at day 3 of ICU treatment), in which MLKL do not regress as usually seen in the cohort (Figure 2), have poor prognosis is certainly very interesting to investigate on possible detrimental functions of persistently elevated MLKL during systemic inflammation and cell death. In this context, it is important to note that induction of apoptosis, measured by increased CK18 serum levels, has been demonstrated to be indicative for patients' prognosis [26]. Our data therefore implicate a previously unknown role of necroptosis, as an important mode of cell death in the context of sepsis diseases. In contrast to apoptosis, which is usually considered as a nonlytic and immunologically silent form of cell death, necroptosis represents a lytic form of cell death, which is described as highly inflammatory, and includes the rapid release of proinflammatory cytokines. Therefore, it seems likely that markers of necroptosis might even be superior to those of apoptosis in the diagnosis of sepsis and estimating the prognosis of those patients.

In this paper, we demonstrate that alterations in MLKL serum levels might be used as a biomarker for patients with critical illness and sepsis, raising the question to the source of MLKL in patients' serum. On one hand, necroptosis leads to cell lysis and a subsequent passive release of intracellular proteins such as MLKL into the serum. On the other hand, next to the function in cell death execution, MLKL is associated with endosomes and controlled the transport of endocytosed proteins, thereby enhancing degradation of receptors and ligands and modulating their induced signaling and facilitating the generation of extracellular vesicles [27]. Moreover, the release of phosphorylated MLKL within extracellular vesicles was suggested to serve as a mechanism for self-restricting the necroptotic activity of this protein. Thus, alterations of circulating MLKL might reflect complex immunological mechanism in sepsis or infectious diseases. Nevertheless, our data on a strong correlation between serum ALT/AST levels and MLKL levels rather argue for a passive release by dying cells, for instance, from the liver [28].

Despite tremendous progresses in the diagnosis and treatment of ICU patients, the triage, diagnostic, and therapeutic management during the first days of treatment still represent a major challenge. The promptness and accuracy of the initial decisions are decisive of the patients' fate as the outcome of, for example, sepsis disease or cardiogenic shock depends on early treatment initiation [5]. In this context, the use of novel biomarkers that allow rapid decision-making with sufficient accuracy may significantly improve the prognosis of critically ill patients [29, 30]. Notably, MLKL serum levels seem to specifically predict the prognosis of patients in the early phase after ICU admission, thus offering a potential novel tool to guide treatment decisions at this critical time point. Given that MLKL at day 3 of ICU treatment is a strong predictor of mortality risk, one could speculate that its use might be implemented into established scoring systems together with markers that detect the initial cause of the critical illness leading to ICU admission (e.g., TWEAK, which has recently been demonstrated to specifically detect sepsis [31]). Notably, the successful implementation of a cell death marker into a clinical prognosis score was recently demonstrated by Bechmann et al., showing that a so-called “CK18 M65-based MELD” score has superior sensitivity and specificity to predict survival of patients with acute liver failure when compared to the MELD score alone [32].

In summary, the data presented here suggest a potential use of MLKL as a tool in the prognostic judgment of critically ill and septic patients during the early phase of their ICU stay. Notwithstanding, these data need to be confirmed in further longitudinal clinical trials using independent cohorts of critically ill patients with and without sepsis before an implementation into clinical algorithms can be considered. Finally, our data imply an important role of MLKL in the molecular pathogenesis of critical illness and should trigger further mechanistic research on the role of MLKL in the regulation of inflammation in this context.

## Figures and Tables

**Table 1:** Study population.

Parameters	All patients
Number	136
Sex (male/female)	76/60
Age, median (range) [years]	66 (18–90)
APACHE-II score, median (range)	18.5 (3–40)
SAPS II score, median (range)	44 (9–80)
ICU days, median (range)	9 (1–137)
Preexisting diabetes, *n* (%)	34.5%
HbA1c [%]	5.9 (4–12.60)
BMI [kg/m^2^]	26.122 (15.9–59.5)
WBC, median (range) [×10^3^/*μ*l]	12.7 (0.1–208)
CRP, median (range) [mg/dl]	122 (<5–230)
Procalcitonin, median (range) [*μ*g/l]	1.0 (0.0–125.2)
Interleukin-6, median (range) [pg/ml]	73 (0–26,000)

APACHE: Acute Physiology and Chronic Health Evaluation; CRP: C-reactive protein; ICU: intensive care unit; SAPS: Simplified Acute Physiology score; WBC: white blood cell.

**Table 2:** Correlations of MLKL serum concentrations at admission day and three days of ICU treatment with other laboratory markers.

	ICU patients			
	MLKL at admission		MLKL at d3	
Parameters	*r*	*p*	*r*	*p*
*Markers of liver function*				
AST	0.279	0.002	0.225	0.039
Bilirubin	0.247	0.004	0.090	0.339
LDH	0.207	0.015	0.266	0.01
*Markers of inflammation*				
CRP	0.028	0.752	0.032	0.766
Procalcitonin	0.148	0.167	0.075	0.526
*Markers of renal function*				
Creatinine	0.229	0.008	0.129	0.220
Urea	0.172	0.046	0.114	0.279
*Others*				
TNF-alpha	0.309	0.044	0.249	0.112
suPAR	0.179	0.042	0.101	0.335
Serum lactate	0.197	0.028	0.088	0.427

*r*: correlation coefficient; *p*: *p* value. *r* and *p* values by Spearman's rank correlation.

## Supplementary Materials

Supplementary Materials Supplementary Figure 1. Serum levels of MLKL in healthy blood donors and patients with different inflammatory or malignant diseases. Serum concentrations of MLKL were analyzed by ELISA in patients with different inflammatory or malignant diseases and compared to healthy blood donors as controls. Supplementary Table 1. Patients' characteristics within low-MLKL and high-MLKL group. Comparison of basal patients' characteristics (sex, presence of sepsis disease, etiology of sepsis disease, severity of sepsis disease, presence of liver cirrhosis, or diabetes mellitus type 2) between the low-MLKL and the high-MLKL group, based on the cutoff of 229.4 pg/ml.
