# Supplementary material for: Elevated Serum Levels of Mixed Lineage Kinase Domain-Like Protein Predict Survival of Patients during Intensive Care Unit Treatment
Source: Dis Markers. 2018 Feb 11;2018:1983421. doi: 10.1155/2018/1983421 (PMC5828132; doi:10.1155/2018/1983421)
Supplement: Supplementary Materials — Supplementary Figure 1. Serum levels of MLKL in healthy blood donors and patients with different inflammatory or malignant diseases. Serum concentrations of MLKL were analyzed by ELISA in patients with different inflammatory or malignant diseases and compared to healthy blood donors as controls. Supplementary Table 1. Patients' characteristics within low-MLKL and high-MLKL group. Comparison of basal patients' characteristics (sex, presence of sepsis disease, etiology of sepsis disease, severity of sepsis disease, presence of liver cirrhosis, or diabetes mellitus type 2) between the low-MLKL and the high-MLKL group, based on the cutoff of 229.4 pg/ml. [file 1983421.f1.pdf]

Supplementary Table 1. Patients´ characteristics within MLKL low and MLKL high group, based on the cut-off of 229.4 pg/ml.

**Sex**		Percent %
<229.4	male	63.0
	female	37.0
>229.4	male	51.3
	female	48.7
**Sepsis**		Percent %
<229.4	yes	72.2
>229.4	yes	64.1
**Etiology of septic disease**		Percent %
<229.4	Sepsis Pulmonary	37.0
	Sepsis Abdominal	13.0
	Sepsis others	22.2
	Cirrhosis	5.6
	Cardiopulmonal	13.0
	Others	7.4
	Acute pancreatitis	1.9
>229.4	Sepsis Pulmonary	38.5
	Sepsis Abdominal	10.3
	Sepsis others	12.8
	Cirrhosis	10.3
	Cardiopulmonal	15.4
	Others	5.1
	Urosepsis	2.6
	Acute liver failure	2.6
	Acute pancreatitis	2.6
**Cirrhosis**		Percent %
<229.4	yes	5.6
>229.4	yes	12.8

Apache-II score		total Number
<229.4	Median	17.5
	Minimum	3
	Maximum	40
>229.4	Median	17
	Minimum	4
	Maximum	31
**Diabetes type II**		Percent %
< 229.4	yes	40.4
> 229.4	yes	31.6

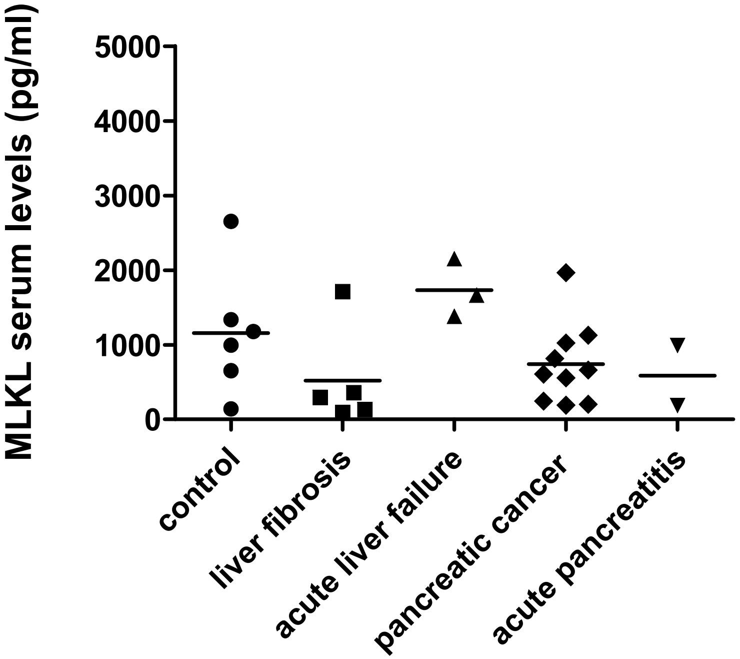

Supplementary Figure 1. Serum levels of MLKL in healthy blood donors and patients with different inflammatory or malignant diseases.
